# Nrf2-mediated anti-oxidant effects contribute to suppression of non-alcoholic steatohepatitis-associated hepatocellular carcinoma in murine model

**DOI:** 10.3164/jcbn.17-125

**Published:** 2018-04-03

**Authors:** Shoji Yamada, Masaki Kimura, Yoshimasa Saito, Hidetsugu Saito

**Affiliations:** 1 Division of Pharmacotherapeutics, Faculty of Pharmacy, Keio University, 1-5-30 Shibakoen, Minato-ku, Tokyo 105-8512, Japan; 2 Division of Gastroenterology and Hepatology, Department of Internal Medicine, Keio University, 35 Shinanomachi, Shinjuku-ku, Tokyo 160-8582, Japan

**Keywords:** hepatocellular carcinoma, gut microbiota, oxidative stress, anti-oxidant effect

## Abstract

The exact mechanisms of hepatocellular carcinoma development in non-alcoholic steatohepatitis remain unclear. In this study, we used a new class of high-fat diet, which could induce hepatocellular carcinoma development without the use of general chemical carcinogens or knockout mice. We investigated the correlation between hepatocellular carcinoma and oxidative stress/anti-oxidant effects after depletion of the gut microbiota by treatment with antibiotics. Mice fed with the steatohepatitis-inducing high-fat diet (STHD-01) for 41 weeks developed hepatocellular carcinoma. Antibiotic-treatment in mice fed with STHD-01 significantly depleted the gut microbiota and significantly ameliorated liver injury/histology. The tumor numbers of hepatocellular carcinoma were dramatically decreased by the antibiotics-treatment. We analyzed the factors involved in oxidative stress and anti-oxidant effects. Oxidative stress was elevated in mice fed with STHD-01, whereas some anti-oxidant factors were significantly elevated after antibiotics treatment. These results suggest that the gut microbiota is a key factor in improving oxidative stress induced by STHD-01 feeding.

## Introduction

The number of patients with non-alcoholic fatty liver disease (NAFLD) has increased in recent years.^(1)^ Dysregulation of adipokines, insulin resistance, and dyslipidemia were reported to induce fat accumulation in the liver of patients with NAFLD.^(2–4)^ Approximately 10% of NAFLD patients develop non-alcoholic steatohepatitis (NASH). Many aspects of NASH pathology are unclear; however, researchers have reported that numerous factors, such as oxidative stress, pathogen-associated molecular patterns (PAMPs)/gut microbiota metabolites, and fatty acid peroxides induce NASH development.^(5)^ Approximately 10% of NASH patients further develop liver cirrhosis and NASH-associated hepatocellular carcinoma (HCC).^(6–8)^ The number of patients with HCC caused by NASH has increased in recent years.^(9–12)^ Additionally, HCC is a common malignant tumor worldwide. However, the mechanisms of NASH-associated HCC are not well-understood.^(1–14)^

There are several types of mice models available for mimicking the condition of HCC in NASH, and they can be divided into two types. One lacks specific genes, such as melanocortin 4 receptor (*Mc4r*), in which high-fat diet (HFD) feeding induces the development of autonomous NASH-associated HCC.^(15)^ The other is a widely used model that uses carcinogens, such as 7,12-dimethylbenz[a]anthracene (DMBA) plus HFD feeding, to induce HCC development in NASH.^(16)^ In the same manner, long-term feeding of mice with HFD can promote the development of HCC in NASH. These models are very useful for investigating the mechanism and natural course of NASH-associated HCC. However, these models also have limitations.^(5,17,18)^

It has been reported that oxidative stress is related to NASH and HCC pathology. Several reports have evaluated the anti-oxidant effects of the microbiome on suppressing HCC development from NASH. For example, *Clostridium butyricum* inhibited liver injury induced by carbon tetrachloride (CCl_4_) via nuclear factor (erythroid-derived 2)-like 2 (Nrf2) activation.^(19)^ In contrast, many types of cancer cells have a mechanism for Nrf2 stabilization; cancer patients showing Nrf2 stabilization have a poor prognosis.^(20)^ In this study, we fed mice with a steatohepatitis-inducing HFD (STHD-01) diet containing various types of fatty acids and cholesterol. This diet reportedly causes mice to develop NASH and NASH-associated HCC in 9 and 36 weeks, respectively.^(21)^ We used a murine model of NASH-associated HCC without the use of carcinogens. The aim of this study was to determine whether Nrf2 and its related anti-oxidant effects prevented or aided the development of NASH-associated HCC in this murine model.

## Materials and Methods

### Animal and histology

SPF C57BL/6J mice were fed a conventional CE-2 diet (CLEA Japan, Inc., Tokyo, Japan) until starting point of 8 weeks of age. The gut microbiota was standardized by mixing the cages every 2–3 days for 2 weeks. Mice were fed the STHD-01 (11% kcal/protein, 72% kcal/fat, and 17% kcal/nitrogen-free extracts; EA Pharmaceuticals Co., Ltd., Tokyo, Japan) for 41 weeks.^(21)^ The control group was fed a standard diet (SD) (AIN-93G; 19% kcal/protein, 12% kcal/fat, and 69% kcal/nitrogen-free extract). Mice were treated with a cocktail of antibiotics (Abx) (1 g/L ceftazidime and 1 g/L metronidazole; both from Sigma-Aldrich, St. Louis, MO) to deplete the gut microbiota. Mice were sacrificed after 41 weeks of feeding with each diet and then feces, plasma, and liver samples were harvested. Histological evaluation was conducted in a blind manner by a hepatologist and two pathologists at Keio University Hospital. Biochemical examination of aspartate aminotransferase (AST) and alanine aminotransferase (ALT) were conducted using a Spotchem EZ (SP-4430, Arkray USA, Inc., Minneapolis, MN).^(22,23)^

### Ethics statement

The use of non-human primates in research. All animal experiments were conducted in accordance with the Institutional Guidelines on Animal Experimentation at Keio University (http://www.animal.med.keio.ac.jp/img/kitei.pdf) and were approved by The Keio University Institutional Animal Care and Use Committee (Permission #13042-(2)). Our animal care and protocols complied with the national guidelines defined by the Science Council of Japan, available at http://www.scj.go.jp/ja/info/kohyo/pdf/kohyo-20-k16-2e.pdf.

EA Pharma Co., Ltd, which is involved in the research and development, manufacturing, and sales of pharmaceuticals was provided the diet of AIN-93G and STHD-01 for all terms. However, with regards to the present manuscript, the authors state that there are no financial, personal, or professional competing interests that might have influenced the performance or presentation of the work in this manuscript.

### Microbiome analysis

At 41 weeks, feces were harvested from the cage of each mouse and resuspended in phosphate-buffered saline (PBS; Wako, Osaka, Japan) (0.1 g/ml). The fecal suspension was crushed using a Bug Crasher (Taitec GM-01, Saitama, Japan) at maximum rotation for 10 min. The sample was incubated on ice for 5 min and centrifuged at 2,300 × *g* at 4°C for 1 min. The DNA pellet was extracted using phenol/chloroform/isoamylalcohol (Thermo Fisher Scientific, Inc., Waltham, MA). Extracted DNA was resuspended in 100 µl Tris/ethylenediaminetetraacetic acid buffer (TE; Sigma-Aldrich) supplemented with 0.5 µl RNase A (Qiagen, Hilden, Germany). The DNA was purified by using the Template Preparation Kit (Roche, Basel, Switzerland).

The obtained DNA was analyzed by terminal restriction fragment length polymorphism analysis (TechnoSuruga Laboratory Co., Ltd., Shizuoka, Japan). The DNA was amplified using fluorescence-labeled primers. The amplified DNA was then treated with the restriction enzyme BS/I (Takara Bio, Inc., Shiga, Japan) and analyzed using the ABI Prism 3130xl DNA Sequencer (Applied Biosystems, Foster City, CA) and Gene Mapper (Applied Biosystems). Cluster analysis was conducted using the GeneMaths program (Applied Maths, Sint-Martens-Latem, Belgium).

To measure the total number of gut bacteria in the feces, a standard curve was generated using genomic DNA from *Escherichia coli* JCM1649T. The sample DNA was amplified with primers 8F (5'-AGAGTTTGATYMTGGCTCAG-3') and 1510R (5'-TACGGYTACCTTGTTACGACTT-3'). Quantitative polymerase chain reaction (qPCR) was conducted by using the SYBR Green PCR Master Mix (Applied Biosystems) and the CFX96Touch (Applied Biosystems). The cycle step was 50°C × 2 min, 95°C × 10 min, (95°C × 30 s, 60°C × 30 s, 72°C × 1 min) × 50 cycles.^(24)^

To identify the bacteria species, the feces were harvested from mice fed STHD-01 treated with Abx. The feces were serially diluted with PBS and disseminated onto brain heart infusion (BHI) broth agar (Honeywell, Inc., Morris Plains, NJ), luria-bertani (LB) agar (Thermo Fisher Scientific, Inc.) + ceftazidime (Sigma-Aldrich), or m-enterococcus agar (Wako). After culture under aerobic conditions at 37°C for 1 or 2 days, individual colonies were picked up, the isolated strains were identified, and 16S ribosomal RNA (rRNA) gene sequences were determined. The 16S rRNA gene was amplified by colony-PCR using Platinum Taq polymerase (Invitrogen, Carlsbad, CA) and 16S rRNA gene-specific primer pairs: 8F (5'-AGAGTTTGATCMTGGCTCAG-3') and 1492R (5'-GGTTACCTTGTTACGACTT-3'). The amplification cycle was as follows: 94°C × 2 min, (94°C × 15 s, 55°C × 30 s, 68°C × 1 min 30 s) × 35 cycles. Each amplified DNA was purified using a QIAquick Gel Extraction kit (Qiagen). Sequence analysis was performed using the BigDye Terminator V3.1 cycle sequencing kit (Applied Biosystems) and Applied Biosystems 3730xl DNA analyzer (Applied Biosystems). The results were compared to sequences in the RDP database and genome database using BLAST to identify similar species.

### Western blotting analysis

Frozen liver tissue samples were homogenized in T-PER solution (Thermo Fisher Scientific, Inc.) containing protease inhibitors (Roche) using a BioMasher (Nippi, Tokyo, Japan). Protein concentration was determined by using the BCA Protein Assay Kit (Thermo Fisher Scientific, Inc.). The homogenized tissue samples were mixed with equal quantities of Laemmli Sample Buffer (Bio-Rad Laboratories, Inc., Hercules, CA) and heated to 95°C for 5 min. The solution was resolved by sodium dodecyl sulfate-polyacrylamide gel electrophoresis (Mini-Protein TGM 7.5%; molecular marker, MagicMark XP Western Protein Standard; Invitrogen). Electrophoresis was performed at 200 V for 45 min. The resolved proteins were then transferred to a nitrocellulose membrane (GE Healthcare Bioscience, Little Chalfont, UK) using a TE 70 semi-dry transfer unit (GE Healthcare Bioscience) at 45 mA for 80 min. The membranes were blocked with blocking buffer (PBS, 0.02% Tween, and 20.5% skim milk) at 4°C overnight. An antibody specific for Nrf2 (1:1,000, Santa Cruz Biotechnology, Inc., Dallas, TX) was used as the primary antibody, while horseradish peroxidase-conjugated anti-rabbit IgG antibody [1:5,000 goat anti-rabbit IgG (H+L); Invitrogen] was used as the secondary antibody. As a control, β-actin-horseradish peroxidase mouse monoclonal IgG (Santa Cruz Biotechnology, Inc.) was used. The membrane was reacted with ECL Western Blotting Detection Reagent (GE Healthcare Bioscience), and protein bands were developed using an ECL mini-camera (GE Healthcare Bioscience). Band density was analyzed using ImageJ software (NIH, Bethesda, MD).

### Quantitative RT-PCR

RNA samples were harvested from the liver using by ISOGEN as previously described.^(25)^ Purified RNAs were reverse-transcribed into cDNA by using the High Capacity cDNA Reverse Transcription Kit (Applied Biosystems), and qPCR was performed using the SYBR Green PCR Master Mix (Applied Biosystems). The results were analyzed by using the CFX96Touch (Applied Biosystems). The cycling steps were as follows: 50°C × 2 min, 95°C × 10 min, (95°C × 30 s, 60°C × 30 s, 72°C × 1 min) × 50 cycles. The primers used are shown in Table 1.

### d-ROMs test

Systemic oxidative stress was analyzed by the d-ROMs test (WISMERLL Co., Ltd., Tokyo, Japan) as reported previously.^(26)^ Briefly, a 10 µl plasma sample was added to pH 4.8 acidity-buffer and mixed. Next, chromogen was added to the buffer and mixed. Finally, the samples were analyzed using a free carpe diem; spectrophotometer (WISMERLL Co., Ltd.).

### BAP test

Oxidant-antioxidant status was analyzed by the BAP test (WISMERLL Co., Ltd.) as reported previously.^(27)^ Briefly, the free carpe diem; spectrophotometer was standardized using water. The buffer was incubated for 10 min at 25°C. Subsequently, 50 µl of chromogen was added to the buffer and mixed. Absorbance of this mixture was measured in the spectrophotometer. Subsequently, 10 µl plasma samples were dissolved in the mixture and incubated on 25°C for 5 min. Sample absorbance was measured in the spectrophotometer.

### Metabolome analysis

Metabolomic analysis was conducted using the Dual Scan package from Human Metabolome Technologies, Inc. (HMT; Yamagata, Japan) using liquid chromatography time-of-flight mass spectrometry (LC-TOFMS) and capillary electrophoresis (CE)-TOFMS for ionic and non-ionic metabolites, respectively, as described previously.^(28,29)^

### Statistical analyses

The results were plotted as the mean ± SE. One-way analysis of variance followed by the Tukey’s post-hoc test was used to compare differences among multiple groups. Student’s *t* test or Mann-Whitney *U* test was used to compare differences between two groups. All comparisons were two-sided, and a *p* value <0.05 was considered significant. All statistical analyses were performed using SPSS 22 for Windows (SPSS, Inc., Chicago, IL).

## Results

### Significant changes of gut microbiota

**Fig. 1:**
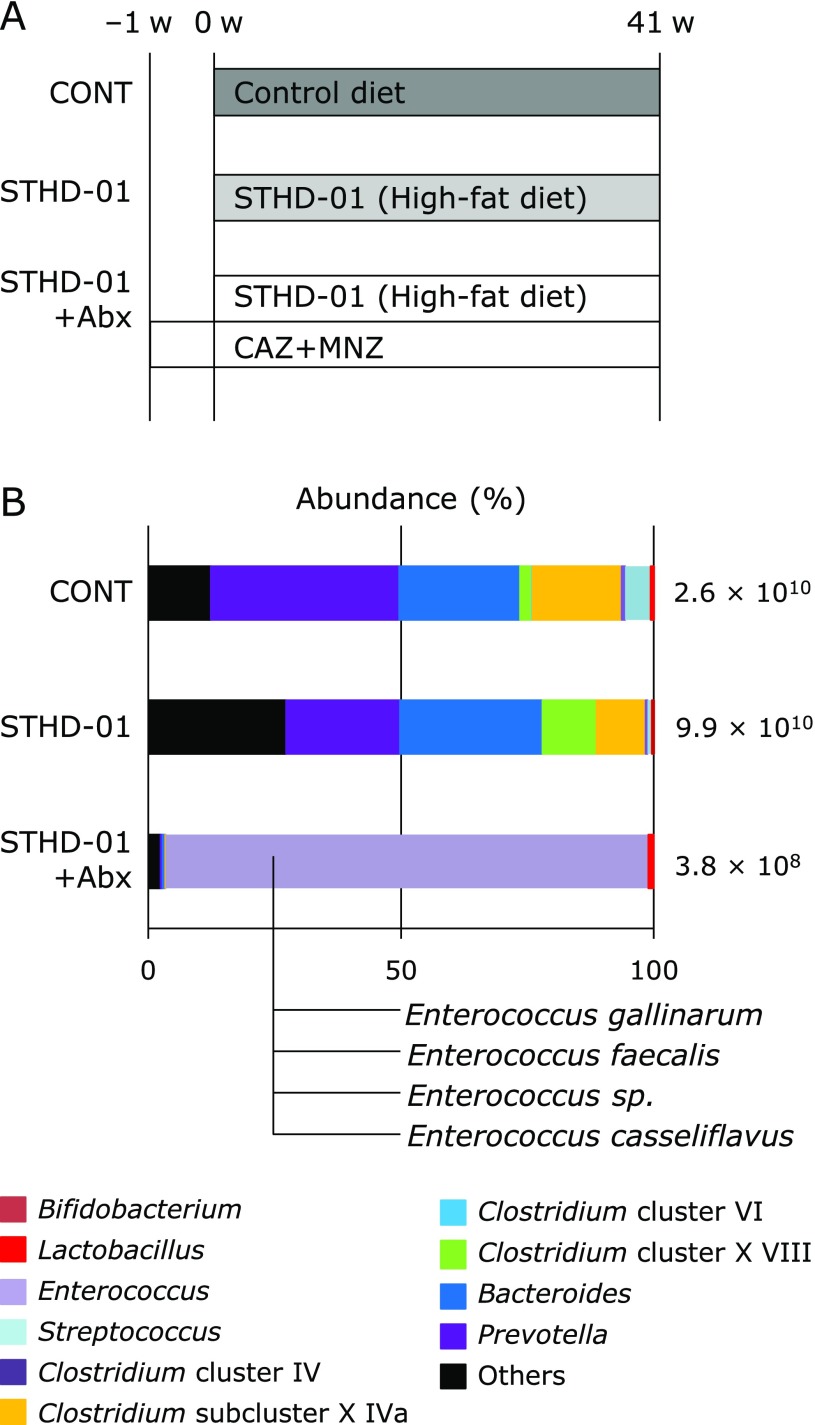
Component of gut microbiota in each group. *Enterococcus* was predominant in the STHD-01 + Abx group. (A) Experimental protocol. (B) Fecal samples were harvested from each mouse (CONT, *n* = 16; STHD-01, *n* = 20; STHD-01 + Abx, *n* = 17) at 41 weeks after STHD-01 feeding. Fecal samples were analyzed by T-RFLP (CONT, *n* = 3; STHD-01, *n* = 3; STHD-01 + Abx, *n* = 4). The abundance of bacterial genera is shown. The total number of bacteria is shown at the right of each column. The under of the column of STHD-01 + Abx indicates the species of bacteria in STHD-01 + Abx.

SPF C57BL/6J mice were fed on a control diet (CONT group), STHD-01 (STHD-01 group), or STHD-01 plus antibiotics (STHD-01 + Abx group) for 41 weeks. The STHD-01 + Abx group was treated with Abx from 1 week before changing the diet (Fig. 1A).

We first examined the gut microbiota in each group. Feeding on STHD-01 did not affect the total number of bacteria, while treatment with Abx decreased the total number of bacteria by more than 1/100-fold. The species of bacteria in the mice of the STHD-01 group was very different from those in the mice of the CONT group. The number of *Bacteroides* and *Clostridium* cluster XVIII was increased. In contrast, number of *Streptococcus*, *Bifidobacterium*, and *Prevotella* was decreased in the STHD-01 group. In the STHD-01 + Abx group, *Enterococcus* was predominant in the gut microbiota. We identified the species of *Enterococcus*. The gut microflora of the mice of the STHD-01 + Abx group comprised of *Enterococcus gallinarum*, *Enterococcus faecalis*, *Enterococcus sp.*, and *Enterococcus casseliflavus* (Fig. 1B).

### Abx treatment significantly reduced HCC numbers

**Fig. 2:**
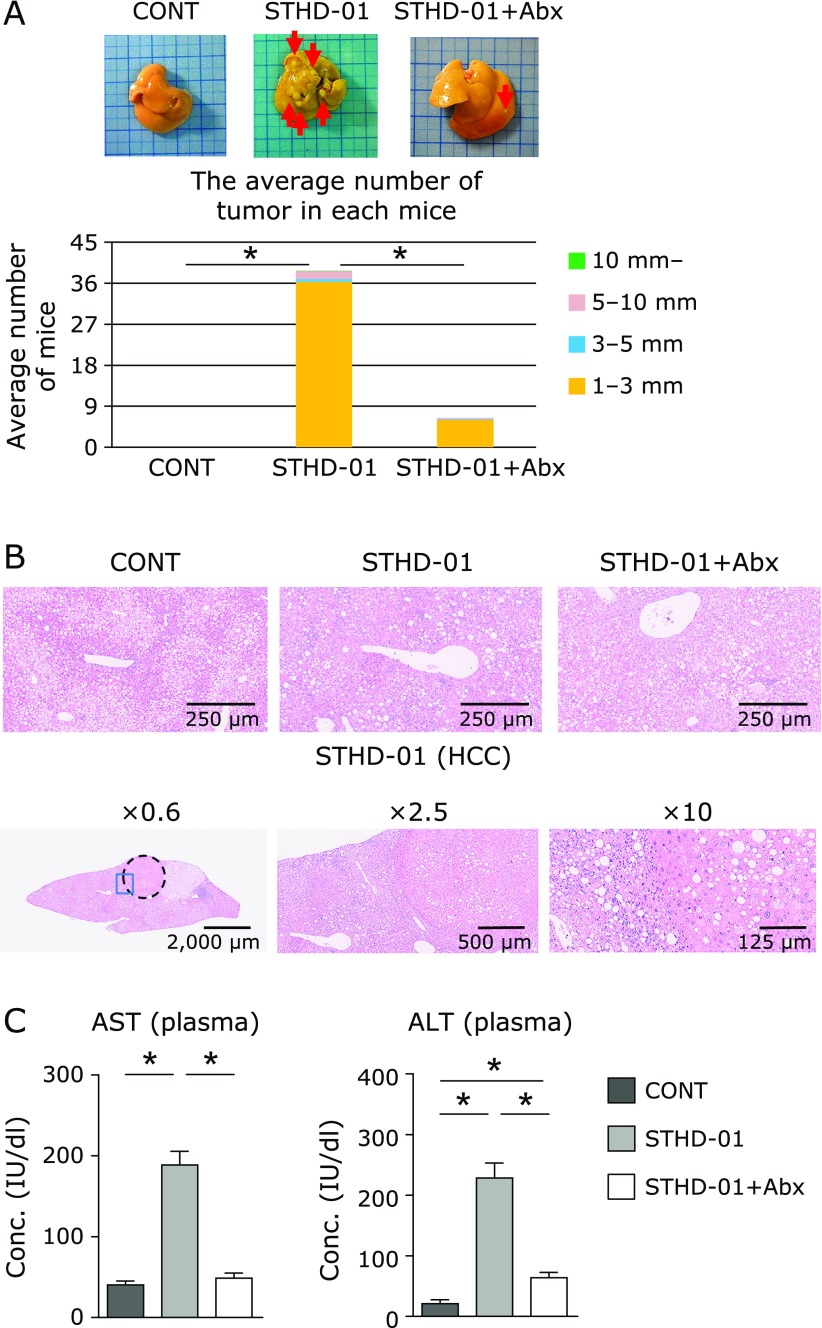
Development of NASH-associated HCC induced by STHD-01 and rescued by antibiotic treatment. (A) Whole liver tissue images (Top), total number of hepatocellular carcinomas (Bottom). (B) Representative liver histology in each group are displayed. (C) The plasma level of aspartate aminotransferase (AST) and alanine aminotransferase (ALT) measured with biochemical test. Data are presented as the mean ± SE (CONT, *n* = 12; STHD-01, *n* = 20; STHD-01 + Abx, *n* = 9). *p<0.001 by Tukey’s test.

Normal C57BL/6J mice fed on STHD-01 developed NASH-associated HCC, which was significantly suppressed by Abx treatment. Liver hematoxylin and eosin (H&E) staining results for each group are shown in Fig. 2. The liver of the mice in the CONT group displayed uniform age-dependent steatosis. The mice of the STHD-01 group showed features of HCC, such as liver cell dysplasia and atypia, and monotonous clear cell changes. The number and features of HCC were higher in the STHD-01 group, and development of HCC was significantly inhibited by treatment with antibiotics (Fig. 2A and B). This indicates that the gut microbiota contributed to hepatocarcinogenesis in this model. The liver injury markers AST and ALT were significantly elevated in the samples from the STHD-01 group and dramatically improved in those from the STHD-01 + Abx group (Fig. 2C).

### Nrf2 and its related antioxidants in the liver

**Fig. 3:**
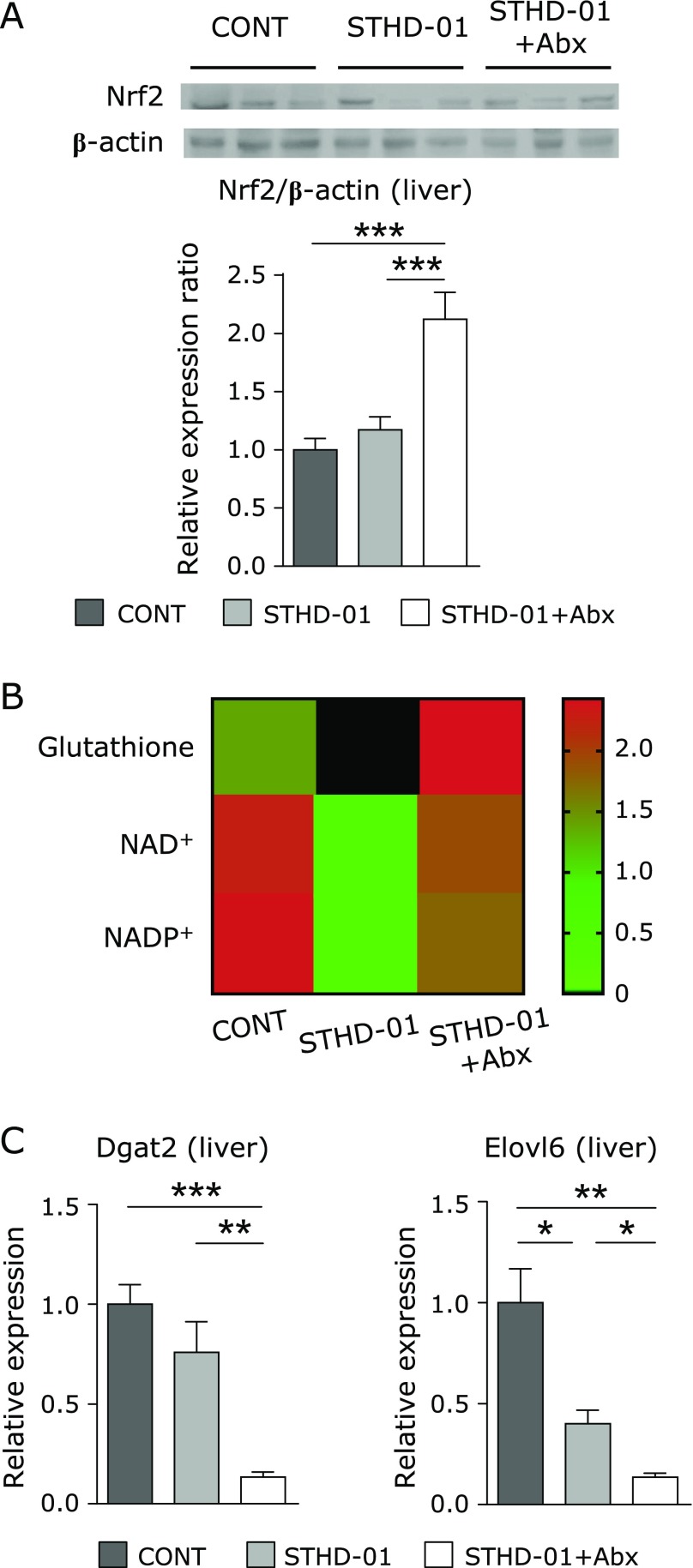
Nuclear factor (erythroid-derived 2)-like 2 (Nrf2) and anti-oxidant metabolomes were elevated by antibiotic treatment. (A) The protein level of Nrf2 is shown. Data are presented as the mean ± SE (CONT, *n* = 8; STHD-01, *n* = 11; STHD-01 + Abx, *n* = 8). (B) Metabolome analysis of the liver in each group. Data are presented as the mean (*n* = 1) (C) TG and fatty acid synthesis related enzymes are shown. Data are expressed as the mean ± SE (CONT, *n* = 5; STHD-01, *n* = 9; STHD-01 + Abx, *n* = 7). *p<0.05, **p<0.01, ***p<0.001 by Tukey’s test.

The Nrf2 expression level in the liver of mice in the STHD-01 + Abx group was significantly elevated compared to that of mice in the other groups (Fig. 3A). The expression of glutathione, a representative anti-oxidant metabolite in the liver, was down-regulated in mice of the STHD-01 group but up-regulated in mice of the STHD-01 + Abx group compared to that in mice of the CONT group (Fig. 3B). The anti-oxidant metabolites nicotinamide adenine dinucleotide (NAD)^+^ and nicotinamide adenine dinucleotide phosphate (NADP)^+^ were reduced in the STHD-01 group, and this effect was reversed by Abx treatment (Fig. 3B).

### Changes in the expression of Nrf2-related enzymes for fat metabolism

Nrf2 was previously shown to regulate fat metabolism.^(28,30,31)^ The expression levels of genes related to triglyceride synthesis and fatty acid elongation, such as diacylglycerol acyltransferase (Dgat) 2 and fatty acid elongase (Elovl) 6, were significantly suppressed in mice of the STHD-01 + Abx group compared to those in mice of the other two groups (Fig. 3C).

### Oxidative stress and anti-oxidants in the plasma

**Fig. 4:**
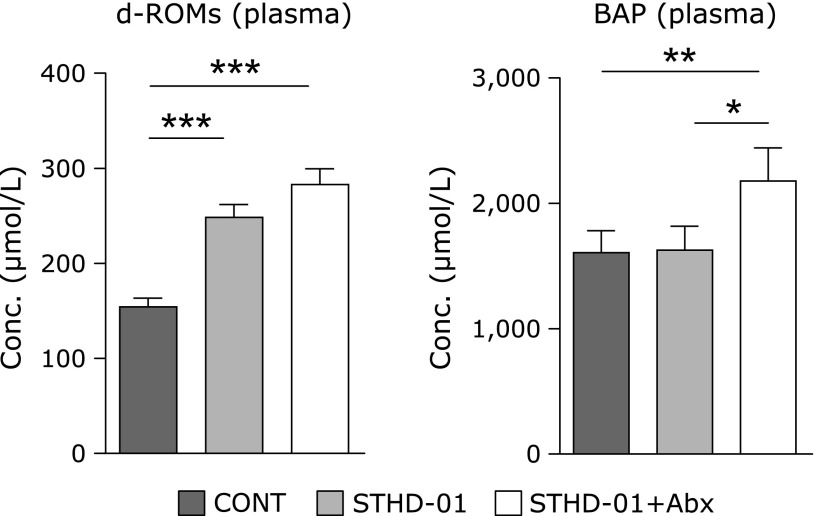
Ability of oxidative stress and anti-oxidative effects in the plasma. Anti-oxidative effects were highest in the STHD-01 + Abx group. The oxidative effect and anti-oxidative effects of plasma were measured in the d-ROMs and BAP tests. Data are shown as the mean ± SE (CONT, *n* = 5; STHD-01, *n* = 9; STHD-01 + Abx, *n* = 7). *p<0.05, **p<0.01, ***p<0.001 by Tukey’s test.

Increased oxidative stress, measured by d-ROMs, in the plasma was observed in the STHD-01 fed groups. In contrast, anti-oxidants measured by BAP in the plasma were dramatically elevated in the STHD-01 + Abx group (Fig. 4). These results indicate that oxidative stress was up-regulated by feeding of STHD-01, while Abx treatment increased the anti-oxidant effects.

## Discussion

Gut microbiota and their metabolites in the pathogenesis of liver diseases have been widely evaluated in recent years. For example, Yoshimoto *et al.*^(17)^ reported that mice fed with a conventional HFD and treated with DMBA developed NASH-associated HCC, which could be treated by the administration of antibiotics. We did not use the tumor initiator DMBA, but demonstrated that HFD was a risk factor of HCC and that a dramatical change in the gut microbiota reduced HCC risk. In our study, gut microbiota showed different characteristics between the three groups (Fig. 1B). Gut microbiota analysis revealed that abundance of the bacterial genera *Bacteroides* and *Clostridium* cluster XVIII increased, while the number of *Streptococcus*, *Bifidobacterium*, and *Prevotella *decreased after feeding with STHD-01. In Abx-treated mice, the number of these bacteria was dramatically decreased in the gut. The gut microbiota of mice in STHD-01 + Abx group was predominantly *Enterococcus*. We identified four species of *Enterococcus*: *Enterococcus gallinarum*, *Enterococcus faecalis*, *Enterococcus sp.*, and *Enterococcus casseliflavus* (Fig. 1B). *Enterococcus* is resistant to chephem antibiotics.^(32)^

We confirmed the similar phenotypes in the mice fed with STHD-01. They only developed NASH-associated HCC, while mice in the STHD-01 + Abx group were resistant to NASH-associated HCC progression.^(17)^ Liver injury markers in the plasma were significantly elevated in the STHD-01 group and decreased in the STHD-01 + Abx group (Fig. 2).

It has been reported that oxidative stress is involved in NASH and HCC pathology.^(33–35)^ Nrf2 expression was significantly up-regulated in the STHD-01 + Abx group. Heat map analysis revealed a decrease in anti-oxidant metabolites, NAD^+^, NADP^+^, and glutathione, in the liver of mice of the STHD-01 group, and this effect was reversed in mice of the STHD-01 + Abx group. A previous study revealed a relationship between the gut microbiota and anti-oxidant effects.^(19)^ Our study also showed that modification of the gut microbiota altered the level of oxidative stress and anti-oxidant effects (Fig. 3A and B).

It has been reported that Nrf2 inhibits lipid metabolism in the liver.^(31,36)^ Another study showed that Nrf2 promoted lipid metabolism.^(30)^ In this study, the expression of genes related to lipid metabolism was suppressed in mice of the STHD-01 + Abx group. Our results indicate that Nrf2 suppressed lipid metabolism and that Nrf2-related gene regulation influences lipid accumulation in the liver of mice of the STHD-01 group and reduces that in mice of the STHD-01 + Abx group (Fig. 3C).

In the STHD-01 + Abx group, the anti-oxidant effect of plasma was elevated. These results were comparable to those of studies showing that increasing of Nrf2 and anti-oxidant metabolites inhibited liver injury and inflammation.^(19)^ Nrf2 and anti-oxidant metabolites in the liver may rescue the pathology of NASH and HCC development by feeding with STHD-01.

The results of this study support that Nrf2 and its related metabolites have protective effects on liver injury, inflammation, and tumorigenesis.^(35,37)^

## Figures and Tables

**Table 1:** Primer sequences

Dgat2	F	TGGGTCCAGAAGAAGTTCCAGAAGTA
	R	ACCTCAGTCTCTGGAAGGCCAAAT
Elovl6	F	CAGCCCCAATGAACATGTCAG
	R	GCGGCTTCCGAAGTTCAAA
Gapdh	F	GTGTCCGTCGTGGATCTGA
	R	CCTGCTTCACCACCTTCTTGA
